# Fibrotic and emphysematous murine lung mechanics under negative-pressure ventilation

**DOI:** 10.1152/ajplung.00087.2024

**Published:** 2025-01-15

**Authors:** K. A. M. Quiros, T. M. Nelson, A. Ulu, E. C. Dominguez, T. M. Nordgren, M. Eskandari

**Affiliations:** 1 Department of Mechanical Engineering, University of California-Riverside, Riverside, California, United States; 2 Division of Biomedical Sciences, Riverside School of Medicine, University of California-Riverside, Riverside, California, United States; 3 Environmental Toxicology Graduate Program, University of California-Riverside, Riverside, California, United States; 4 BREATHE Center, School of Medicine, University of California-Riverside, Riverside, California, United States; 5 Department of Environmental and Radiological Health Sciences, Colorado State University, Fort Collins, Colorado, United States; 6 Department of Bioengineering, University of California-Riverside, Riverside, California, United States

**Keywords:** biomarkers, compliance, COPD, negative pressure, pulmonary mechanics

## Abstract

Chronic obstructive pulmonary disease (COPD) is the third leading cause of death worldwide, and the progressive nature heightens the calamity of the disease. In existing COPD studies, lung mechanics are often reported under positive-pressure ventilation (PPV) and extrapolations made from these studies pose restrictions as recent works have divulged disparate elastic and energetic results between PPV and more physiological negative-pressure ventilation (NPV) counterparts. This nonequivalence of PPV and NPV must be investigated under diseased states to augment our understanding of disease mechanics. To assess the comparability of diseased pulmonary mechanics in PPV and NPV, we pose a novel study to parse out the currently entangled contributions of ventilation mode and diseased state by analyzing murine PV curves from porcine pancreatic elastase (PPE) and hog dust extract (HDE) induced COPD models under positive and negative pressures. We find that, for PPE-exposed, under NPV, volume, compliance (*C*, *C_start_*, and *C_def_*), and hysteresis are increased in diseased states and that under PPV, only compliance (*C* and *C_start_*) is increased. For HDE-exposed, under NPV, volume, compliance (*C*, *C_inf_*, *C_def_*, and *K*), and hysteresis are decreased, whereas, under PPV, only volume and static compliance decreased. All significant mechanical variations due to disease were observed solely at higher pressures (40 cmH_2_O) under both PPV and NPV. Our nuanced conclusions indicate the detection capabilities of multiple mechanics-based biomarkers are sensitive to the ventilation mode, where NPV exhibits more altered mechanics metrics in PPE-exposed and HDE-exposed groups compared with PPV counterparts, suggesting the resolution of biomarkers when applied under NPV research considerations may offer greater versatility.

**NEW & NOTEWORTHY:**

We evaluate whether ubiquitous pressure-volume (PV) curve biomarkers depend on the ventilation mode under which they were collected (i.e., positive- or negative-pressure ventilation). This is a significant investigation considering recent works have revealed PV curves are distinct and noninterchangeable under the two ventilation modes. Multiple biomarkers noted under negative-pressure ventilation are lacking from positive-pressure counterparts, albeit for small-scale species considerations. Future investigations should confirm the applicability of these findings for large-scale specimens for clinical considerations.

## INTRODUCTION

Approximately 545 million individuals have a chronic respiratory disease and, annually, 3.2 million deaths are caused by chronic obstructive pulmonary disease (COPD) alone, making respiratory disease a leading cause of morbidity and mortality worldwide (1, 2). COPD encompasses several pathologies including emphysema, chronic bronchitis, and small airway disease (e.g., inflammation and fibrosis), with root causes including occupational (e.g., organic and inorganic dusts, and fumes), environmental (e.g., air pollution), and lifestyle (e.g., smoking) exposures (3–7). These pathologies are characterized by changes to distal airways and parenchyma, which cause detrimental mechanical alterations that restrict breathing (8, 9).

COPD and the associated lung mechanics have been studied and reported in animal models under positive-pressure ventilation (PPV) (10–12) and in clinical studies under both mechanical ventilation (i.e., invasive and noninvasive PPV) (13, 14) and spontaneous breathing (i.e., spirometry) (1, 15). Prevalent noninvasive clinical studies advance COPD diagnostics and safe ventilation strategies, but understanding the base mechanical changes to the isolated diseased lung can help clarify the contributions of lung structure (16, 17). Isolation of lung mechanics is often accomplished with ex-vivo PPV; however, this is known to be nonphysiological—particularly when compared with negative-pressure ventilation (NPV), which more closely mimics diaphragm contraction—and the results may mistranslate when applied to spontaneous breathing (1, 18–20). Although much has been gained by PPV, emerging studies validate this point and call into question the comparability of lung mechanics under PPV and NPV (21–24), even demonstrating disparities in common biomarkers—parameters widely used to comprehend alterations caused by disease—such as static and dynamic compliances between the two modes (21, 24). This underscores the necessity to dissect the currently intertwined mechanisms of the diseased state and ventilation mode responsible for pressure-volume (PV) curve alterations to evaluate how PPV mechanical features fair against the mechanics of NPV.

To address this question, this study utilizes an established custom-designed ventilation device to perform positive- and negative-pressure inflation testing on excised mouse lungs with two separate forms of late-stage COPD (emphysema and inflammation-associated peribronchial fibrosis) and compare results with proven PPV findings (25, 26). Established pulmonary disease models (3, 12, 27, 28) were analyzed with a range of lung organ-level mechanics (quasi-static compliance, starting compliance, inflation compliance, deflation compliance, Salazar–Knowles *K*, viscoelastic relaxation, hysteresis, and energy loss) (17, 26, 29–32).

First, emphysema was modeled via a standard elastase exposure as previously performed (33, 34), which has been studied extensively for PPV (12); this allows rigorous comparisons between available PPV literature and our current PPV/NPV study. In this model, the porcine pancreatic elastase introduced into the lung results in the progressive destruction of tissues simulating emphysema (12). Second, we expand this question to separately investigate how lung mechanics are altered due to chronic dust exposure. This organic dust exposure causes chronic inflammation resulting in small airway disease (i.e., peribronchial fibrosis) and COPD (35, 36); this exposure model is vastly understudied (3, 27). Investigating and understanding the mechanics behind these various avenues of disease manifestation and progression are pertinent to the development of vital early diagnosis techniques (3, 16, 37, 38).

Both mouse COPD models are used to compare prevailing PPV biomarkers within the existing literature to PPV and NPV findings of this current work, with special attention given to PV metrics recently noted to vary between the two ventilation modes (21, 24). Ultimately this study determines if mechanics found to differ between healthy and disease in PPV are applicable and present in NPV; the insights from this study illuminate whether ventilation mode or diseased-state mechanisms and dependencies command biomarker behaviors.

## MATERIALS AND METHODS

### Mouse Models

Twenty male C57BL/6 mice weighing 29.4 ± 3.5 g (8–12 wk old, Jackson Laboratory, Bar Harbor, ME) were separated into four unique treatment groups—two exposed groups coupled with two control groups—to independently assess the pulmonary mechanics of two different models of COPD: elastase-induced and chronic-dust exposure. Outlined exposures *Groups I* to *IV* followed previously established methods and protocols to endorse desired disease manifestations (27, 33): [*Group I*: porcine pancreatic elastase-exposed (PPE-exposed)] emphysema was induced (*n* = 5) via a single intranasal instillation of porcine pancreatic elastase (0.9 U) and euthanized 4 wk postexposure as previously performed and validated via mean linear intercept method (33, 34); [*Group II*: age-matched control for *Group I* (PPE-control)] the emphysema control group received (*n* = 5) a single instillation of 1X phosphate-buffered saline (PBS) and aged for 4 wk. [*Group III*: hog dust extract exposed (HDE-exposed)] Chronic inflammation and resulting peribronchial fibrosis were induced (*n* = 5) via intranasal exposure to 12.5% hog dust extract (HDE) (vol/vol) thrice weekly for 21 wk as previously performed and validated via Ashcroft’s score (27, 34). [*Group IV*: age-matched control for *Group III* (HDE-control)] The HDE-control group (*n* = 5) intranasally received 1X PBS thrice weekly for 21 wk. Mice were anesthetized with isoflurane for procedures and monitored and housed under a 12:12-h light-dark cycle with ad-libitum access to food and water. All the experiments and procedures were approved by the University of California, Riverside’s Institutional Animal Care and Use Committee [IACUC; protocol #20200014; some control mice were also part of a previous analysis (24, 26) and then utilized in (34)].

### Pulmonary Function

Mice were euthanized on the day of testing by isoflurane overdose, after which trachea cannulation and lung inflation to 0.5 mL followed to prevent atelectasis. The heart-lung bloc was removed and stored in PBS. Exposure and euthanasia protocols were performed by a separate set of individuals than mechanical testing and analysis to ensure measurements and analysis were done in a blind manner. Mechanical testing was performed by cyclic inflation-deflation and was conducted by using our established custom electromechanical ventilation apparatus, as previously described (25, 26, 39). During testing, real-time measurements of transpulmonary pressure and lung expansion volume (not just applied volume) were collected (25, 26). The inflation protocol was as follows: initial 5 cmH_2_O preload, three preconditioning cycles, one test cycle, and one inflation ramp preceding a 120 s hold. To investigate known pressure and frequency dependencies (26, 29, 31, 40–42), two peak pressures (20 and 40 cmH_2_O ± 15% with a volume-control apparatus) (40), each at two cycling frequencies [5 and 20 breaths per minute (BPM)] were tested. This protocol was completed under PPV and NPV as previously and extensively described where, briefly, NPV was conducted by applying a negative pressure to the lung until the targeted lung pressure was reached (25, 26).

**Figure 1.**
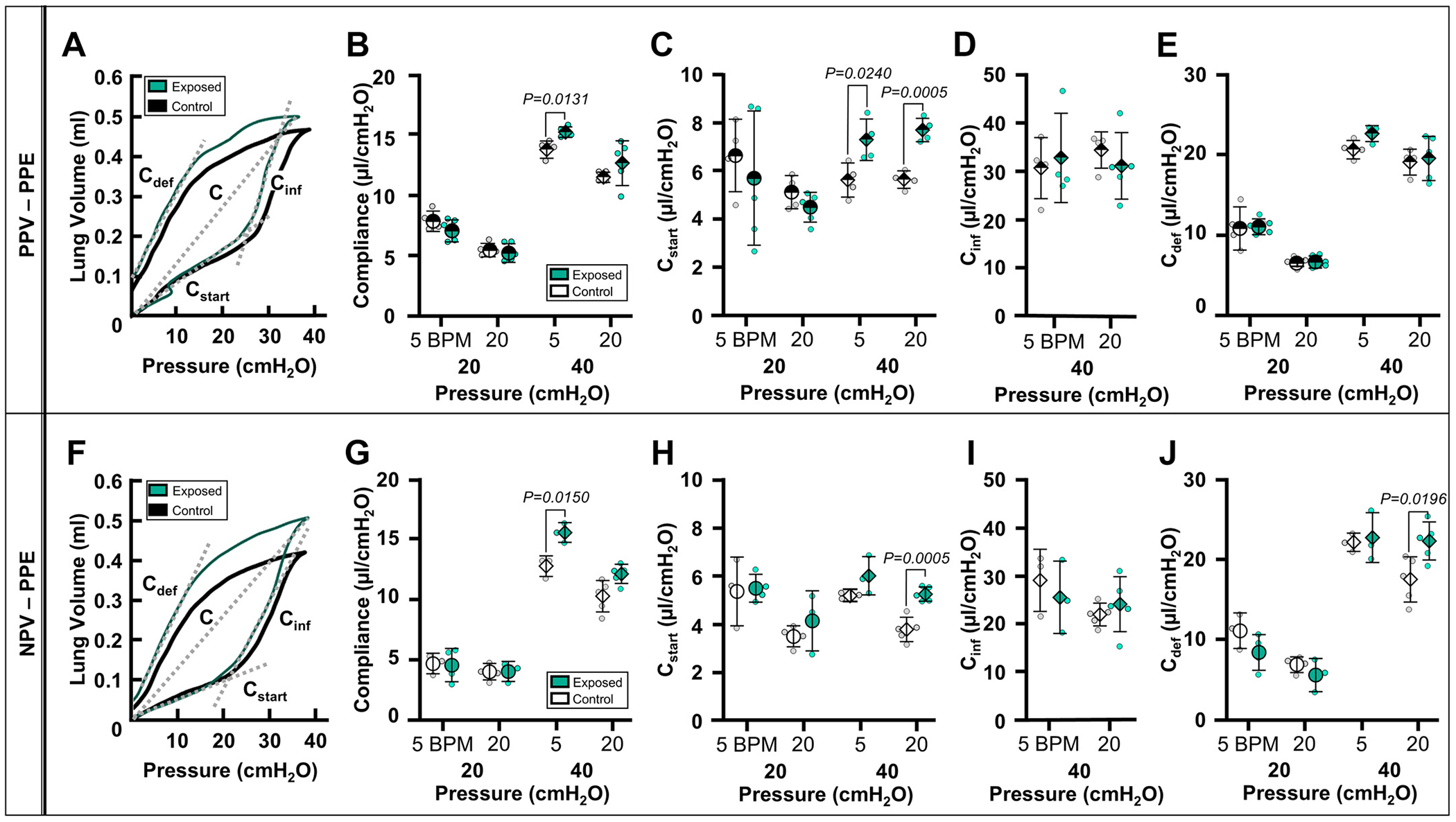
Representative PV curves demonstrating upward shifts of PPE-exposed (green) curves under PPV (*A*) and NPV (*F*). Means ± SD of extracted metrics from PPE-exposed (green) and PPE-control (white) subjects are reported for PPE treatment group at 5 and 20 BPM and 20 (circle) and 40 cmH_2_O (diamond) under PPV (half-filled) and NPV (solid): quasi-static compliance (*B* and *G*), starting compliance (*C* and *H*), inflation compliance (*D* and *I*), and deflation compliance (*E* and *J*). BPM, breaths per minute; NPV, negative-pressure ventilation; PPE, porcine pancreatic elastase; PPV, positive-pressure ventilation; PV, pressure-volume.

To quantify the structural alterations induced by pulmonary disease (28), the elastic and energetic measures of pressure-volume (PV) curves were analyzed. Two promising biomarkers, as identified in the literature as deflation compliance (*C_def_*) and quasi-static compliance (*C*), were calculated and analyzed (18, 19, 43). *C* was calculated as the ratio of peak-lung volume to peak pressure, where peak and plateau pressures were assumed equal due to slow inflation rates (17, 44). Other compliance measures, starting compliance (*C_start_*), inflation compliance (*C_inf_*), and deflation compliance (*C_def_*), were also calculated as tangents to the PV curve in their respective locations by using linear regression, as described previously (Fig. 1A) (26, 29). To compare alternative analysis methods, the Salazar–Knowles equation was also used to quantify the deflation section of the PV curve (45):

(1) $$\text{V}=A-B{\text{e}}^{-K\text{P}}$$

where *V* is volume, *P* is pressure, and *A*, *B*, and *K* are constants.

Lung volume (*V*) was recorded for each test and analyzed. Viscoelastic alterations to the tissue were quantified as the percentage drop in measured pressure during the constant load (120 s) (30). Two energetic measures, hysteresis and energy loss, were calculated and analyzed as the area encapsulated by the PV curve and the normalized area, respectively (Fig. 4A) (46, 47).

### Bronchoalveolar Lavages

Bronchoalveolar lavage fluid (BALF) was collected and centrifuged at 1,500 rpm for 5 min. Cells from three washes were combined (total 3 mL PBS). Differential cell counts were performed for each mouse where 300 cells were counted for the number of macrophages, eosinophils, neutrophils, and lymphocytes.

### Statistical Analysis

The student’s t-test was performed to compare values between exposed and control groups (unpaired) with *P* = 0.05 declared as the threshold for significance (48) (Version 9.1.0, GraphPad Software, San Diego, CA). Occasionally, lungs failed to reach the target pressure and these trials were discarded.

## RESULTS

### Compliances and Lung Volume

Effects of emphysema were demonstrated by a leftward shift of the PV curve of the PPE-exposed group compared with the PPE-control group (Fig. 1, A and F) (49). Figure 1, B–E and G–J, reports static and dynamic compliances for PPE-control (white) and PPE-exposed (green) groups under PPV and NPV, respectively. Under PPV, the alterations to the PV curve of the PPE-exposed lungs were manifested as increased C at 40 cmH_2_O at 5 BPM and increased *C_start_* at 40 cmH_2_O at 5 and 20 BPM when compared with the PPE-control group (Fig. 1, B and C). Similarly, under NPV, *C* (40 cmH_2_O at 5 BPM), *C_start_* (40 cmH_2_O at 20 BPM), and *C_def_* (40 cmH_2_O at 20 BPM) increased in PPE-exposed lungs (Fig. 1, G, H, and J).

**Figure 2.**
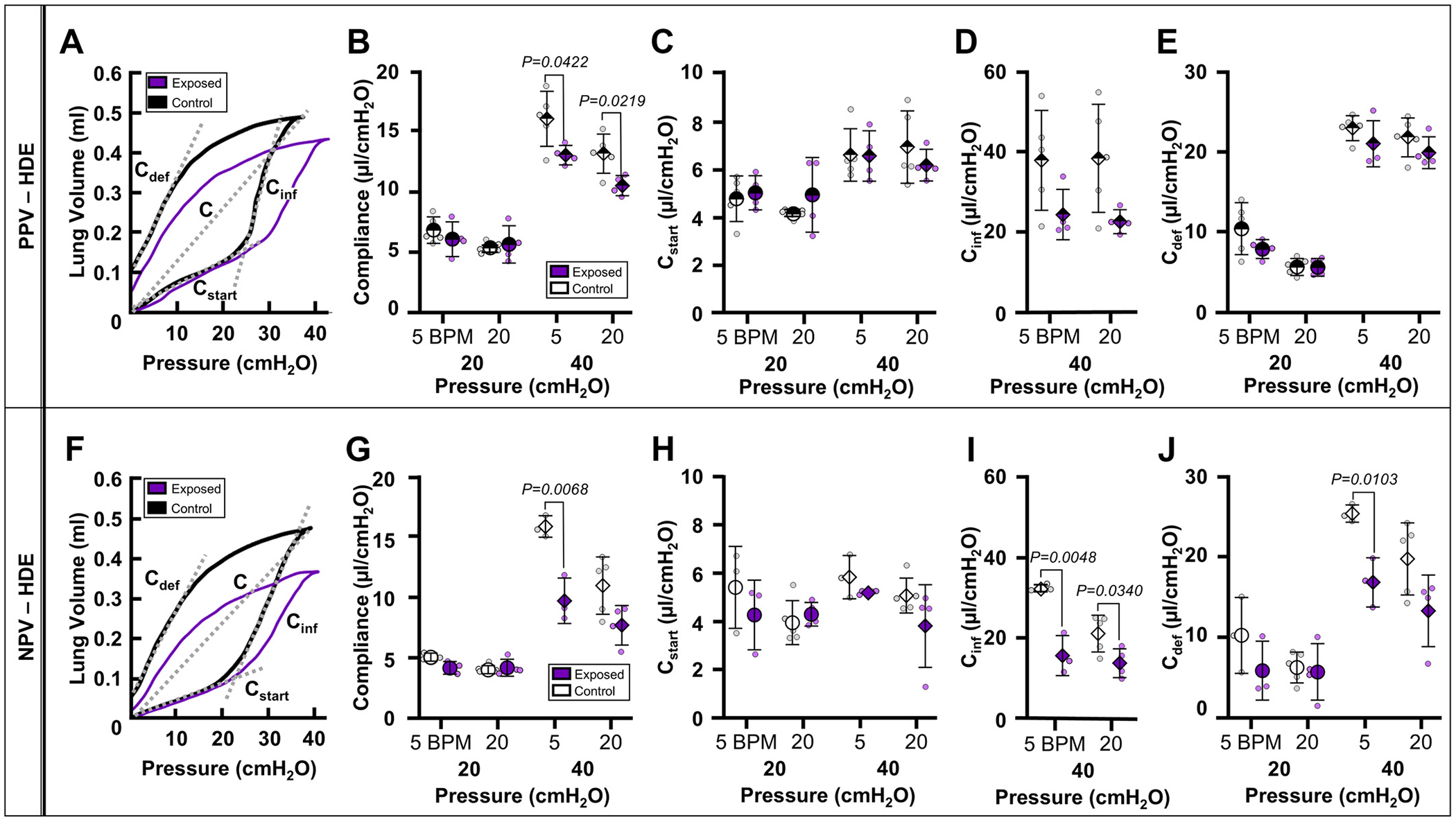
Representative PV curves demonstrating downward shifts of HDE-exposed (purple) curves under PPV (*A*) and NPV (*F*). Means ± SD of extracted metrics from HDE-exposed (purple) and HDE-control (white) subjects are reported for HDE treatment group at 5 and 20 BPM and 20 (circle) and 40 cmH_2_O (diamond) under PPV (half-filled) and NPV (solid): quasi-static compliance (*B* and *G*), starting compliance (*C* and *H*), inflation compliance (*D* and *I*), and deflation compliance (*E* and *J*). BPM, breaths per minute; HDE, hog dust extract; NPV, negative-pressure ventilation; PPV, positive-pressure ventilation; PV, pressure-volume.

HDE also altered the lung PV response resulting in differed compliance measures between HDE-control (black) and HDE-exposed (purple) (Fig. 2, A and F). Specifically, in PPV, *C* decreased at 40 cmH_2_O at 5 and 20 BPM (Fig. 2B), whereas in NPV, *C* (40 cmH_2_O at 5 BPM), *C_inf_* (40 cmH_2_O at 5 and 20 BPM), and *C_def_* (40 cmH_2_O at 5 BPM) were reduced (Fig. 2, G, I, and J) for the HDE-exposed group compared with the respective HDE-control group.

**Figure 3.**
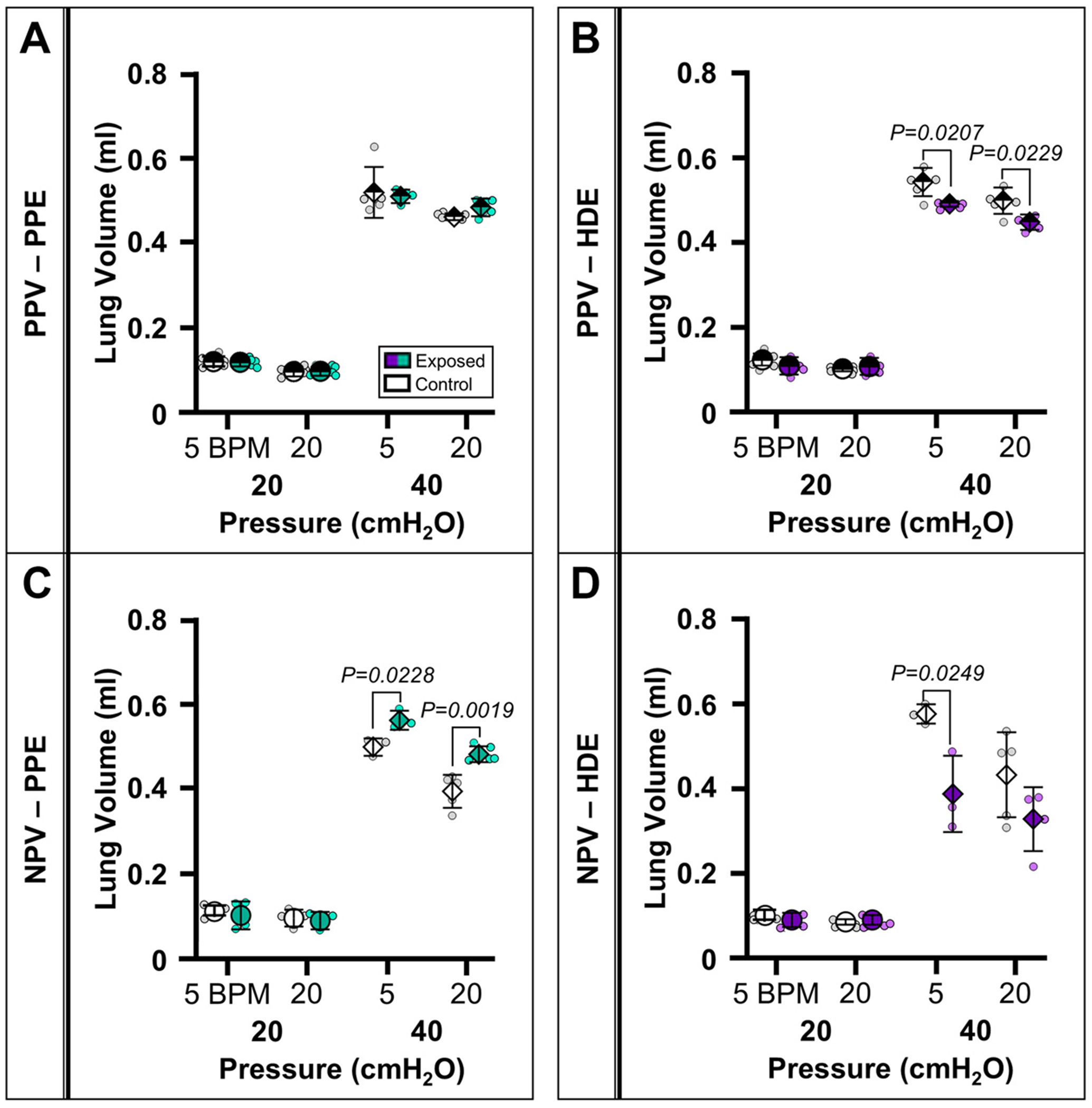
Means ± SD of lung volume for PPE (*A* and *C*) and HDE (*B* and *D*) treatment groups comparing control (white) and exposed (green/purple) groups at 20 (circle) and 40 cmH_2_O (diamond) under PPV (*A* and *B*) and NPV (*C* and *D*). HDE, hog dust extract; NPV, negative-pressure ventilation; PPE, porcine pancreatic elastase; PPV, positive-pressure ventilation; PV, pressure-volume.

Figure 3 reports the resultant *V* for PPE (Fig. 3, A and C, PPV and NPV respectively) and HDE (Fig. 3, B and D, PPV and NPV respectively) groups. Under PPV, the peak-lung volume did not vary between PPE-exposed and PPE-control lungs; moreover, volume was reduced at 40 cmH_2_O at 5 and 20 BPM in HDE-exposed lungs under PPV. Under NPV, PPE-exposed lungs reported increased *V* at 40 cmH_2_O at 5 and 20 BPM compared with PPE-control subjects and HDE-exposed lungs demonstrated reduced *V* at 40 cmH_2_O at 5 BPM compared with HDE-control subjects.

**Figure 4.**
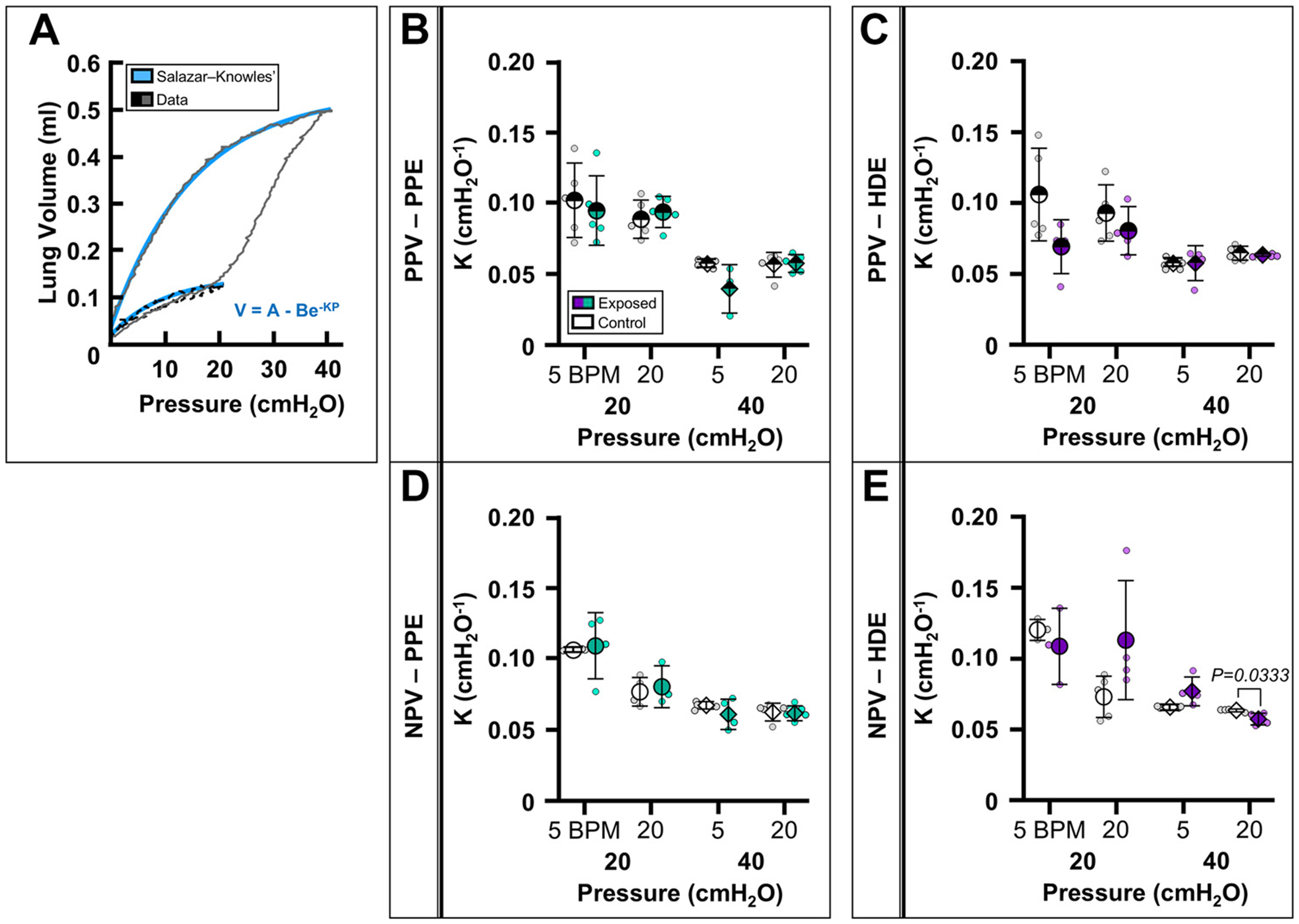
*A*: PV curve (20 cmH_2_O, black dashed line; 40 cmH_2_O, grey solid line) fitted with Salazar–Knowles equation (blue) to calculate parameter *K*. Means ± SD of *K* for PPE (*B* and *D*) and HDE (*C* and *E*) treatment groups at 5 and 20 BPM comparing control (white) and exposed (green/purple) groups at 20 (circle) and 40 cmH_2_O (diamond) under PPV and NPV. BPM, breaths per minute; HDE, hog dust extract; NPV, negative-pressure ventilation; PPE, porcine pancreatic elastase; PPV, positive-pressure ventilation; PV, pressure-volume.

Figure 4A demonstrates the goodness of fit of the Salazar–Knowles model (blue) for PV curves collected at 20 (black dashed line) and 40 cmH_2_O (grey solid line). Figure 4, B and D, shows that *K* detected no clear differences between the PPE-exposed and PPE-control groups under PPV nor NPV. For the HDE groups, *K* was lower in HDE-exposed specimens for 40 cmH_2_O at 20 BPM under NPV alone (Fig. 4D).

### Energetics

**Figure 5.**
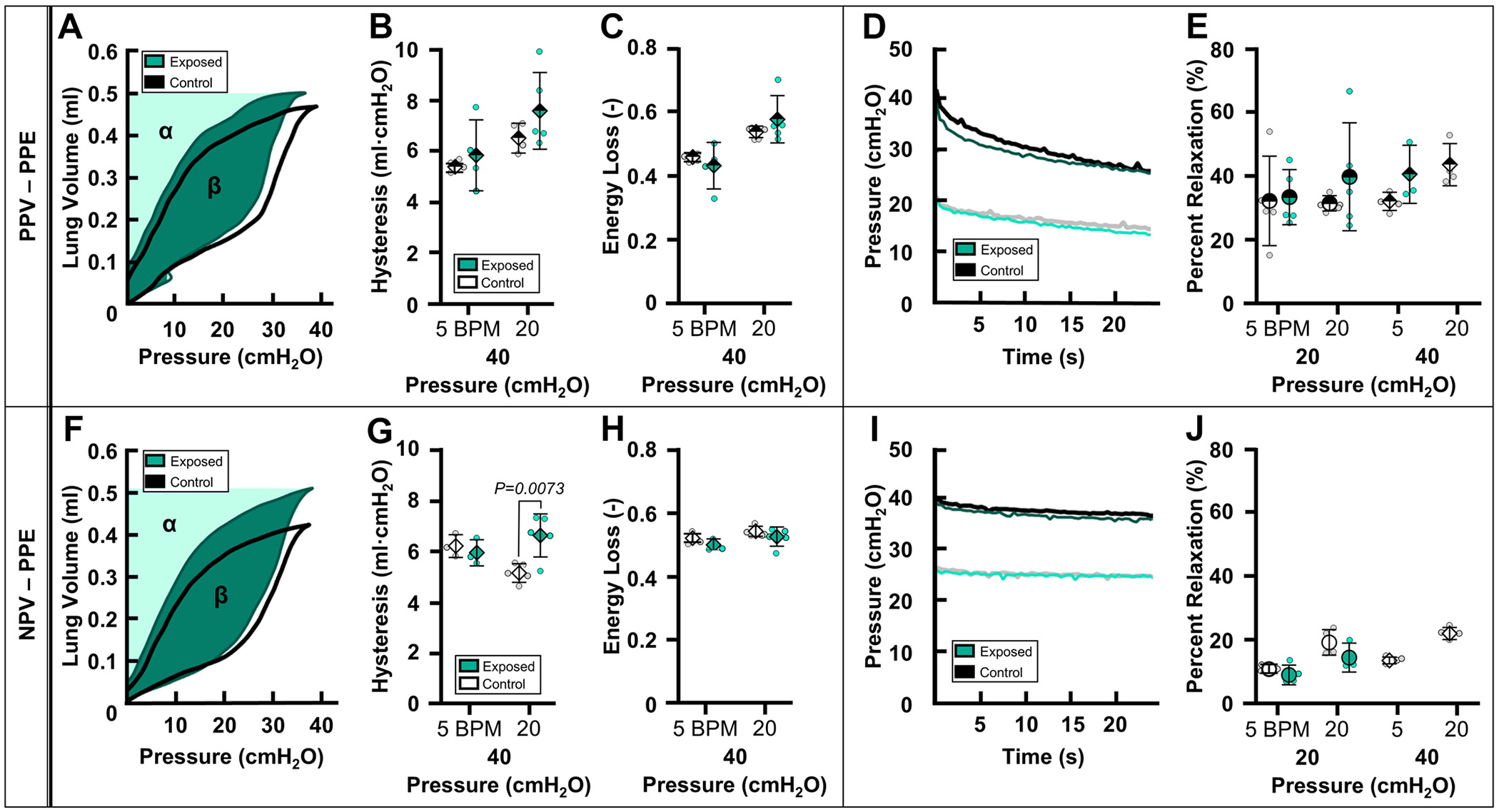
PV curves demonstrating hysteresis (β) and energy loss $\left(\frac{\text{β}}{\text{β+α}}\right)$ of PPE-control (black) compared with PPE-exposed (green) subjects under PPV (*A*) and NPV (*F*). Means ± SD of resulting mechanics comparing PPE-control (white) and PPE-exposed (green) groups under PPV (half-filled) and NPV (solid): hysteresis (*B* and *G*) and energy loss (*C* and *H*) at 40 cmH_2_O. D and I: viscoelastic relaxation of a representative specimen demonstrating comparison of percent relaxation curves of PPE-control (black) and PPE-exposed (green) subjects. *E* and *J*: percent relaxation at 20 (circle) and 40 cmH_2_O (diamond). NPV, negative-pressure ventilation; PPE, porcine pancreatic elastase; PPV, positive-pressure ventilation; PV, pressure-volume.

Energetic measures including hysteresis and energy loss are reported in Fig. 5, A–C and F–H, for PPE groups under PPV and NPV, respectively. At 40 cmH_2_O and 20 BPM, hysteresis was greater for the PPE-exposed group than the PPE-control group solely under NPV (Fig. 5G). Energy loss did not vary between PPE-exposed and PPE-control groups under PPV nor NPV (Fig. 5, C and H).

**Figure 6.**
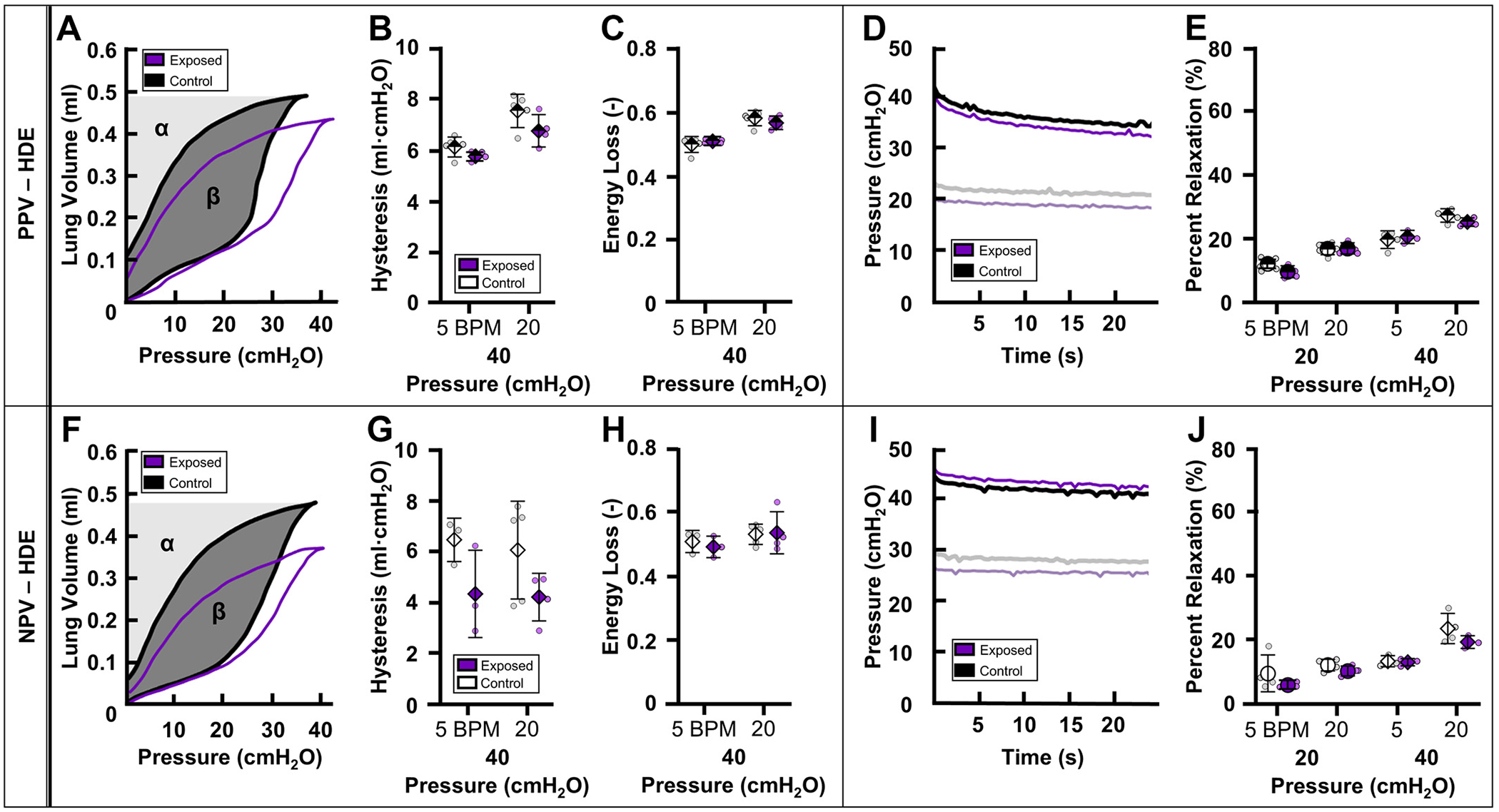
PV curves demonstrating hysteresis (β) and energy loss $\left(\frac{\text{β}}{\text{β+α}}\right)$ of HDE-control (black) compared with HDE-exposed (purple) subjects under PPV (*A*) and NPV (*F*). Means ± SD of resulting mechanics comparing HDE-control (white) and HDE-exposed (purple) groups under PPV (half-filled) and NPV (solid): hysteresis (*B* and *G*) and energy loss (*C* and *H*) at 40 cmH_2_O. *D* and *I*: viscoelastic relaxation of a representative specimen demonstrating comparison of percent relaxation curves of HDE-control (black) and HDE-exposed (purple) subjects. *E* and *J*: percent relaxation at 20 (circle) and 40 cmH_2_O (diamond). HDE, hog dust extract; NPV, negative-pressure ventilation; PPV, positive-pressure ventilation; PV, pressure-volume.

Although the HDE-exposed group showed decreased hysteresis compared with HDE-control specimens, the difference was not significant (Fig. 6G). Energy loss did not vary between HDE-exposed and HDE-control groups (Fig. 6, C and H).

### Viscoelastic Relaxation

Figure 5, D and I, demonstrates representative relaxation curves for PPE groups under PPV and NPV, respectively. Similarly, Fig. 6, D and I, demonstrates representative relaxation curves of HDE groups under PPV and NPV, respectively. Under PPV and NPV, relaxation did not vary between exposed and control groups for PPE or HDE (Fig. 5, E and J, and Fig. 6, E and J, respectively).

### Bronchoalveolar Lavages

Differential cell counts from collected BALF showed total cell counts (*P* = 0.0029, total cell count, HDE-exposed) and macrophages (*P* = 0.0312, macrophages, HDE-exposed) were elevated in both the PPE- and HDE-exposed groups and neutrophils were significantly higher in the HDE-exposed group (*P* = 0.0123, neutrophils, HDE-exposed). Eosinophils and lymphocytes did not vary between either exposed or control group.

Reported values for lung volume, quasi-static compliance, starting compliance, inflation compliance, deflation compliance, Salazar–Knowles *K*, viscoelastic relaxation, hysteresis, and energy loss under PPV and NPV can be found in Table 1 and Table 2, respectively.

## DISCUSSION

This study seeks to determine the commanding mechanisms (i.e., ventilation mode or diseased state) responsible for PV curve alterations in diseased lungs ventilated with positive and negative pressures. To that aim, results of biomarkers in HDE- and PPE-exposed groups collected under NPV are compared with PPV counterparts and existing PPV results from the literature to determine if biomarkers are sensitive to the mode of ventilation. This novel study was motivated by recent renewed debates regarding PPV versus NPV, where studies suggest that mechanics are dependent on inflation mode (21–23, 50–53). Here, negative-pressure PV curves of exposed groups demonstrated similar behavior to well collected and documented shifts in PV curves from PPV (Table 3) (20). However, a greater number of significant biomarker alterations are noted here under NPV than our PPV counterparts. Specifically, at 40 cmH_2_O, for PPE-exposed, static and starting compliance (*C* and *C_start_*) are increased in diseased states under NPV and PPV but under NPV alone, volume, deflation compliance (*C_def_*), and hysteresis are also increased. Correspondingly, for HDE-exposed, volume and static compliance are decreased in diseased states under NPV and PPV but under NPV alone, inflation and deflation compliance (*C_inf_* and *C_def_*), and *K*, are additionally decreased in disease. Neither exposure group reports significant differences at 20 cmH_2_O (Table 1 and Table 2). The observed differences at high pressures were anticipated as disparities between healthy and diseased subjects within the literature are often reported at higher pressures (Table 3) (18, 19).

Interestingly, within the literature, each previously studied biomarker that was noted here to be altered solely under NPV has been observed to be altered due to disease under PPV in at least one study. In other words, although in this current study we do not observe the same biomarker differences under PPV that we observe under NPV, other studies have documented each of these significances under various PPV studies (see Table 3). For example, we do not observe significant volume alterations in PPE-exposed under PPV despite Limjunyawong et al. (18), Devos et al. (20), and Hantos et al. (54) observing significant volume changes in elastase-induced emphysema under PPV; here this volume alteration is observed in only our NPV study. The inability for PPV to recreate the totality of multiple NPV biomarker significances is noteworthy and may indicate that NPV is more sensitive to the physical alterations that accompany disease.

In the following sections, we contrast our resulting NPV mechanics to our PPV counterparts and existing PPV results from the literature, summarized in Table 3, to discuss the potential underlying physiological basis for any differences or lack thereof. Unfortunately, chronic-dust exposure models are not well studied and articles reporting the lung mechanics of these diseased mice were unavailable at the time of this manuscript’s publication. However, this exposure model has been demonstrated to result in fibrotic regions in the lung (27), which aligns with the mechanics reported here, as such, the HDE-exposed mice are compared with other fibrosis models (i.e., bleomycin).

### Inflation Mechanics

Under NPV, *C*, *C_start_*, *C_inf_*, and *V* were altered in exposed groups, where specifically, *C*, *C_start_*, and *V* increased in PPE-exposed, whereas *C*, *C_inf_*, and *V* decreased in HDE-exposed groups compared with the respective control groups (Figs. 1 and 2). These *C*, *C_start_*, and *V* trends are similarly demonstrated here in our PPV counterparts and or found in PPV global lung mechanics studies (Table 3) (20, 28, 54). Furthermore, this restricted expansion—lower *V*—is demonstrated in PPV digital image correlation (DIC) studies of murine lungs, which find reduced surface strains in HDE-exposed lungs compared with control groups (37). The mechanisms responsible for these alterations in NPV are likely identical to those suggested in PPV studies. Specifically, the decreased *C* and *V* noted in HDE-exposed lungs are caused by excessive collagen deposits that restrict lung expansion (27, 34, 43) and the increased *C* and *V* in PPE-exposed occur due to the loss of septal walls—characteristic of emphysema—which increases internal volume and eases expansion (4, 12).

For inflation mechanics, the comparative results between our NPV findings and PPV counterparts appear to inconsistently reflect the disparities reported within the literature between the two ventilation modes. For example, *C_inf_* has been shown to be ventilation mode-dependent (i.e., lower under NPV compared with PPV) and here is seen to be sensitive to HDE exposure under NPV but not PPV (24). Similarly, *C_start_* is thought to be less ventilation mode-dependent and here we observe agreement between PPV and NPV in PPE-exposed. Although this indicates that ventilation mode heavily influences potential biomarker behavior, not all metrics analyzed here reflect that finding. Particularly, quasi-static compliance has been shown to be lower under NPV compared with PPV in murine subjects (i.e., ventilation mode-dependent) but here is sensitive to both exposures under both modes (24). This conflicting applicability of healthy PPV versus NPV results demonstrates that ventilation mode dependence in healthy animals alone is not enough to fully predict which potential disease biomarkers may be affected by ventilation methodologies.

This puts into perspective the limitations of other literature, which examines ventilation mode dependencies. Including prior studies of healthy porcine lungs which find inflation pathways differ between ventilation modes and find surface strains resulting from NPV are lower and more evenly distributed than those reported in PPV, despite expansion to identical lung volumes (21). Special attention should be given to reevaluating ventilation mode dependencies in different disease models, which has briefly been explored (23, 55). The results presented here are additional indicators that these ventilation mode dependencies are not thoroughly understood. Although they are currently thought to be caused by disparate levels of recruitment, gas decompression in peripheral alveoli, and or energy dissipation in parenchymal tissues which may be affected by disease (21, 56).

*C_start_* indicates airway compliance in degassed lungs and is altered in PPV-tested diseased states, suggesting damaged airway structures (57). In non-degassed diseased subjects, however, *C_start_* has not previously been investigated and is thought to reflect the number of naturally open “lung units” (i.e., level of aeration) at end-expiration in healthy lungs. This means that *C_start_* is less reflective of alterations to tissue compliance and is rather more reflective of aeration state (29, 58). Therefore, the results of this study, increased *C_start_* in emphysema (i.e., PPE-exposed) under both PPV and NPV, may arise due to the characteristic destruction of septal walls, which increases the amount of open air spaces at end-expiration, thereby increasing the starting compliance (49). Furthermore, this observed mechanical trend appears to quantitatively measure the known increases in aeration levels demonstrated by computed tomography (CT) images in mouse models of elastase-induced emphysema and human clinical studies, which similarly occurs due to increased air-space enlargement in emphysema (59, 60).

### Deflation Mechanics

*K* and *C_def_* both quantify the compliance of the deflation portion of the PV curve but are calculated differently and are thus influenced by different factors (28, 29, 45). *K*, for instance, is fundamentally uninfluenced by volume, as described by the Salazar–Knowles model (Fig. 4), whereas *C_def_* is influenced by lung volume as the slope of the PV curve near end-expiration (Fig. 1, A and F, and Fig. 2, A and F) (28, 29, 45). As such, both measures of deflation compliance were analyzed in this study to assess, which is more reliable for disease detection under NPV. We find that these two measures of deflation compliance do not agreeably identify disease in either the HDE-exposed or the PPE-exposed group, and we propose the different calculation methods are likely the reason for the discrepancy. Furthermore, here neither *K* nor *C_def_* is altered in either disease model under PPV.

Specifically, *C_def_* demonstrates biomarker behavior—significant differences between control and exposed—for both disease models under NPV but solely at high pressures (40 cmH_2_O) (Figs. 1J and 2J). However, in this study, *K* was not altered in PPE-exposed under PPV or NPV in direct disagreement with numerous PPV studies that report *K* is reduced in elastase-induced emphysema models (Fig. 4, Table 3); this indicates *K* may be ventilation mode-dependent, which has not previously been demonstrated; however, again, we could not replicate *K* alterations seen in the literature under PPV (Fig. 4) (19, 28). For our HDE model, *K* does report biomarker behavior at 40 cmH_2_O, indicating a stiffer lung in the HDE-exposed group (Fig. 4). Detectable stiffening in HDE-exposed was anticipated as chronic dust exposure induces chronic inflammation and subsequently fibrosis [as previously verified via Ashcroft’s score (27)], where the characteristic collagen buildup indicative of fibrosis restricts lung expansion (27, 37, 61). These results of HDE-associated fibrosis agree with *K* results reported with other fibrosis models (28, 34, 62).

Interestingly, *K* counterintuitively indicated softer tissue compliance in the HDE-exposed group at lower pressure/higher frequency and higher pressure/lower frequency despite indicating stiffer tissue behavior at higher pressure/higher frequency, as expected (28, 34, 62). To explain this apparent inconsistency, we propose the decrease in *K* observed in this study is due to the mechanisms of strain stiffening at higher frequencies and higher pressures as opposed to overall tissue stiffening (26, 63, 64). This proposition is emboldened by the fact that the disagreement between frequencies occurs at 40 cmH_2_O, where, at this pressure, most alveoli are recruited and the lung has expanded past the stretch limitations of the elastin, engaging the less-extensible and stop-length collagen fiber response (4, 65, 66). It is worth noting that the divergent and unexpected results indicating a softer lung were not present in the compliance measure of *C_def_* but unique to *K*.

Under NPV, *C_def_*, which has demonstrated ventilation mode dependence between PPV and NPV (21, 24), in this study portrays behavior in line with previous positive-pressure investigations, where *C_def_* increased in PPE-exposed and decreased in HDE-exposed groups but did not vary here under PPV (Fig. 1, E and J, and Fig. 2, E and J) (18, 19). The inability to recreate PPV findings may be the result of the ventilation mode dependency of *C_def_* or could indicate that PPV is less reliable at detecting disease via PV curves.

### Energetics

The detailed contributions of lung hysteresis during inflation/deflation testing are not well understood (67). In isolated tissues, hysteresis is ascribed to ECM structural properties such as fiber concentrations, friction between fibers, and tissue viscoelasticity (34, 68–70). In lung inflation tests, noted hysteresis indicates additional contributions of surfactant, alveolar recruitment/decruitment, and the network behavior of individually inflating/deflating alveoli (71, 72). Whether or not ventilation mode is also a contributor to hysteresis is unclear.

The ventilation mode dependence of hysteresis has recently been investigated with divergent results (i.e., hysteresis was lower in NPV as compared with PPV in porcine lungs but slightly elevated in NPV as compared with PPV in murine lungs) (21, 24). In this study, trends from PPV and NPV tests are not comparable despite agreement between prior PPV emphysema studies and the NPV test reported here (Figs. 5 and 6) (Table 3) (28). This inconsistency again makes it difficult to decipher if any potential ventilation mode dependence is affecting the mechanics responsible for hysteresis in diseased states. The reasoning behind this increased hysteresis in diseased states is not entirely clear. In emphysema, increased hysteresis is likely connected to the altered recruitment mechanisms associated with the emphysematous lung due to septal wall destruction (49, 59).

### Confirmation of Disease State

To confirm disease states, the BALF from each lung was collected and a differential cell count was performed. This analysis was conducted to allow further confirmation of the disease in addition to our prior publications, which demonstrate the success of these exposure models through the appropriate histology and morphological analysis (e.g., mean linear intercept and Ashcroft’s score) (27, 33). Differential cell counts from collected BALF endorsed diseased states as results—elevated total cell counts, macrophages, and neutrophils (HDE-exposed only)—align with reports from the literature (12, 27, 28, 33, 73).

### Limitations

The connection of the results presented herein to the literature is done tentatively as differences in protocols (e.g., ventilatory apparatus, testing procedure, model type, and implementation) limit the comparability as partially described in Table 3. The results from this study may have diagnostic relevance but is worth clarifying that PV curves have not been considered a viable diagnostic tool for some time and that their usage has limitations (74). However, the potential of PV curves to be used as supplemental diagnostic tools should not be ignored, given the documented potential and the need for improved diagnostic strategies (75, 76) as spirometry and CT have inherent flaws (e.g., cost, reliability, and sensitivity) (15, 77). It is also worth noting that the ventilator apparatus we use most closely resembles a dual limb circuit and results from a single limb or passive circuit may differ. We recognize that employing quasi-static ex-vivo testing limits the assessment of physiological contributions (e.g., perfusion, gas exchange, and surfactant generation), given that the lung is removed fromits environment; however, this trade-off enables the evaluation of elastic properties by isolating effects fromflow resistance and the chest cavity (17, 54, 63), although the contribution of the latter is relatively small in C57BL/6 mice (78). Throughout this study, chronic-dust exposure fibrosis is compared within the severely limited body of literature to partially relevant available models (i.e., bleomycin) (27). Although comparisons between PPE- and HDE-exposed groups would have been interesting, the utilized diseasemodels required different aging periods and age is known to alter lung mechanics (79). This study used all male mice, given the dependence of sex on lung mechanics, particularly from COPD subjects, findings need to be confirmed in female mice (80). Thus, the results reported here should not be assumed to be identical for female subjects. In addition, although pressures were meticulously monitored, the use of a volume-controlled apparatus resulted in unavoidable slight deviations in plateau pressures. Finally, leaks are known to be inevitable, and occasionally inflation tests failed to reachmaximumpeak pressures within an acceptable range and these subjects’ results were not reported. Unfortunately, this decreased the number of subjects analyzed and affected significances.

## Figures and Tables

**Table 1.** Means ± SD of lung volume, quasi-static compliance, starting compliance, inflation compliance, deflation compliance, K, percent relaxation, hysteresis, and energy loss in PPE and HDE exposure groups under PPV

Positive-Pressure Ventilation								
Treatment Group	PPE				HDE			
Peak pressure, cmH_2_O	20		40		20		40	
Frequency, BPM	5	20	5	20	5	20	5	20
Lung volume, mL								
Control	0.12 ± 0.01	0.10 ± 0.01	0.52 ± 0.06	0.46 ± 0.01	0.12 ± 0.02	0.10 ± 0.01	0.54 ± 0.03	0.50 ± 0.03
Exposed	0.12 ± 0.01	0.10 ± 0.01	0.51 ± 0.02	0.49 ± 0.02	0.11 ± 0.02	0.11 ± 0.02	0.49 ± 0.01*	0.45 ± 0.02*
*C*, μL/cmH_2_O								
Control	7.94 ± 0.85	5.54 ± 0.54	13.87 ± 0.71	11.58 ± 0.43	6.88 ± 1.10	5.41 ± 0.46	16.13 ± 2.29	13.24 ± 0.16
Exposed	7.13 ± 0.91	5.30 ± 0.77	15.32 ± 0.43*	12.75 ± 0.18	6.12 ± 1.44	5.69 ± 0.16	13.12 ± 0.81*	10.58 ± 0.84*
*C_start_*, μL/cmH_2_O								
Control	6.63 ± 1.52	5.10 ± 0.70	5.60 ± 0.72	5.62 ± 0.37	4.75 ± 0.94	4.13 ± 0.19	6.56 ± 1.08	6.89 ± 1.50
Exposed	5.68 ± 2.82	4.46 ± 0.63	7.29 ± 0.87*	7.70 ± 0.49*	5.0 ± 0.71	4.91 ± 1.54	6.52 ± 1.04	6.14 ± 0.65
*C_inf_*, μL/cmH_2_O								
Control			30.89 ± 6.32	34.63 ± 3.78			37.27 ± 9.66	38.57 ± 1.35
Exposed			33.01 ± 9.25	31.35 ± 6.91			25.89 ± 5.92	22.80 ± 2.98
*C_def_*, μL/cmH_2_O								
Control	10.91 ± 2.7	6.59 ± 0.7	20.71 ± 1.17	19.17 ± 1.61	10.56 ± 3.23	5.80 ± 1.06	23.06 ± 1.56	21.92 ± 2.42
Exposed	11.10 ± 0.99	6.73 ± 0.75	20.19 ± 6.03	19.64 ± 2.76	8.02 ± 1.18	5.75 ± 1.10	21.13 ± 2.87	20.02 ± 2.00
*K*, cmH_2_O^−1^								
Control	0.103 ± 0.026	0.089 ± 0.013	0.046 ± 0.026	0.057 ± 0.009	0.106 ± 0.032	0.093 ± 0.020	0.057 ± 0.004	0.065 ± 0.005
Exposed	0.095 ± 0.025	0.094 ± 0.011	0.030 ± 0.024	0.058 ± 0.006	0.069 ± 0.019	0.080 ± 0.017	0.058 ± 0.012	0.063 ± 0.001
Percent relaxation, %								
Control	32.0 ± 14.0	31.3 ± 2.39	31.9 ± 2.87	43.4 ± 6.62	12.1 ± 1.96	16.9 ± 1.90	19.7 ± 2.80	27.3 ± 2.12
Exposed	33.2 ± 8.62	39.6 ± 16.9	40.4 ± 9.07		9.58 ± 1.90	16.9 ± 1.78	20.6 ± 2.10	25.2 ± 1.33
Hysteresis, mL·cmH_2_O								
Control			5.43 ± 0.23	6.55 ± 0.59			6.16 ± 0.40	7.60 ± 0.66
Exposed			5.88 ± 1.40	7.62 ± 1.50			5.79 ± 0.20	6.81 ± 0.65
Energy loss, %								
Control			45.9 ± 1.2	53.8 ± 1.8			49.8 ± 2.5	58.1 ± 2.4
Exposed			43.1 ± 7.3	57.7 ± 7.4			50.8 ± 7.3	56.6 ± 2.1

* *P* < 0.05 vs. control.

HDE, hog dust extract; PPE, porcine pancreatic elastase; PPV, positive-pressure ventilation; SD, standard deviation.

**Table 2.** Means ± SD of lung volume, quasi-static compliance, starting compliance, inflation compliance, deflation compliance, K, percent relaxation, hysteresis, and energy loss in PPE and HDE exposure groups under NPV

Negative-Pressure Ventilation								
Treatment Group	PPE				HDE			
Peak pressure, cmH_2_O	20		40		20		40	
Frequency, BPM	5	20	5	20	5	20	5	20
Lung volume, mL								
Control	0.11 ± 0.01	0.09 ± 0.02	0.52 ± 0.08	0.40 ± 0.04	0.087 ± 0.023	0.085 ± 0.006	0.52 ± 0.098	0.43 ± 0.089
Exposed	0.101 ± 0.029	0.089 ± 0.0167	0.564 ± 0.031*	0.484 ± 0.017*	0.088 ± 0.012	0.087 ± 0.010	0.405 ± 0.062*	0.304 ± 0.072
*C*, μL/cmH_2_O								
Control	4.73 ± 0.70	4.05 ± 0.60	14.9 ± 4.04	10.3 ± 1.12	4.42 ± 1.22	4.10 ± 0.30	14.40 ± 3.10	11.03 ± 2.11
Exposed	4.59 ± 1.20	4.096 ± 0.68	15.53 ± 0.62*	12.20 ± 0.72	4.27 ± 0.37	4.22 ± 0.55	11.05 ± 1.96*	7.31 ± 1.55
*C_start_*, μL/cmH_2_O								
Control	5.37 ± 1.17	3.50 ± 0.38	4.51 ± 1.65	3.79 ± 0.45	5.39 ± 1.20	3.94 ± 0.81	5.49 ± 0.88	5.06 ± 0. 64
Exposed	5.51 ± 0.50	4.14 ± 1.02	6.18 ± 0.63	5.26 ± 0.27*	4.48 ± 1.09	4.41 ± 0.44	5.97 ± 0.99	3.30 ± 1.66
*C_inf_*, μL/cmH_2_O								
Control			43.0 ± 25.9	22.0 ± 2.12			30.06 ± 6.58	21.33 ± 4.09
Exposed			27.47 ± 6.30	24.15 ± 5.14			19.69 ± 5.69*	13.19 ± 3.19*
*C_def_*, μL/cmH_2_O								
Control	11.2 ± 1.81	6.95 ± 0.84	20.6 ± 3.51	17.6 ± 2.52	9.18 ± 3.85	6.29 ± 1.71	23.40 ± 4.43	19.82 ± 4. 02
Exposed	8.48 ± 1.92	5.67 ± 1.69	23.72 ± 2.74	22.37 ± 2.12*	5.33 ± 2.78	4.87 ± 3.21	21.92 ± 6.46*	12.02 ± 4.35
*K*, cmH_2_O^−1^								
Control	0.106 ± 0.0003	0.077 ± 0.0087	0.058 ± 0.019	0.062 ± 0.006	0.119 ± 0.006	0.073 ± 0.013	0.066 ± 0.002	0.063 ± 0.001
Exposed	0.11 ± 0.02	0.081 ± 0.012	0.061 ± 0.009	0.062 ± 0.005	0.116 ± 0.022	0.126 ± 0.041	0.080 ± 0.010	0.056 ± 0.004*
Percent relaxation, %								
Control	11.4 ± 0.84	19.7 ± 3.46	14.05 ± 0.93	22.49 ± 1.62	9.56 ± 5.00	17.05 ± 9.85	20.95 ± 13.20	27.85 ± 9.24
Exposed	9.50 ± 2.67	14.94 ± 3.71	40.87 ± 5.13	64.31 ± 9.66	5.74 ± 0.74	9.94 ± 1.39	25.01 ± 14.66	30.73 ± 13.98
Hysteresis, mL·cmH_2_O								
Control			6.96 ± 2.37	5.18 ± 0.33			6.02 ± 1.32	6.05 ± 1.72
Exposed			5.81 ± 0.46	6.65 ± 0.76*			4.87 ± 1.27	3.94 ± 0.89
Energy loss, %								
Control			55.7 ± 8.0	54.3 ± 1.4			0.514 ± 0.025	0.535 ± 0.028
Exposed			0.500 ± 0.013	0.526 ± 0.027			0.549 ± 0.069	0.559 ± 0.064

* *P* < 0.05 vs. control.

HDE, hog dust extract; PPE, porcine pancreatic elastase; NPV, negative-pressure ventilation; SD, standard deviation.

**Table 3.** Summary of findings and methods from this work (boldface) and PPV murine lung mechanics studies in the literature

	Treatment		Findings^†^		Strain	Sex	Ventilation Mode	Peak Inflation, mL or cmH_2_O	Rate	Reference
Emphysema studies	PPE	*V* increased	*C_def_* increased		C57BL/6	M	PPV	35 cmH_2_O	3 mL/Min	Limjunyawong et al. (18)
	PPE	*C_def_* increased	*K* decreased		BALB/c	M/F	PPV	35 cmH_2_O	2 cmH_2_O/s	Robichaud et al. (19)
	PPE	*C* no variation	*V* increased		BALB/c	M	PPV	30 cmH_2_O	3.75 cmH_2_O/s	Devos et al. (20)
	PPE	*C* no variation	*K* decreased	Hysteresis increased	BALB/c	M	PPV	30 cmH_2_O	3.75 cmH_2_O/s	Vanoirbeek et al. (28)
	PPE	*V* increased			CBA/Ca	F	PPV	20 cmH_2_O	~1 cmH_2_O/s	Hantos et al. (54)
	**PPE**	***C* increased**	***C_start_* increased**		**C57BL/6**	**M**	**PPV**	**20 and 40 cmH2O**	**5 and 20 BPM**	**This work**
	**PPE**	***C* increased** ***V* increased**	***C_start_* increased** **Hysteresis increased**	***C_def_* increased**	**C57BL/6**	**M**	**NPV**	**20 and 40 cmH_2_O**	**5 and 20 BPM**	**This work**
Fibrosis studies	Bleomycin	*C_def_* decreased	*V* decreased		C57BL/6	F	PPV	35 cmH_2_O	3 mL/min	Limjunyawong et al. (18)
	Bleomycin	*C* decreased	*V* decreased		BALB/c	M	PPV	30 cmH_2_O	3.75 cmH_2_O/s	Devos et al. (20)
	Bleomycin	*C* decreased	*K* decreased	Hysteresis increased	BALB/c	M	PPV	30 cmH_2_O	3.75 cmH_2_O/s	Vanoirbeek et al. (28)
	**Chronic dust**	***C* decreased**	***V* decreased**		**C57BL/6**	**M**	**PPV**	**20 and 40 cmH_2_O**	**5 and 20 BPM**	**This work**
	**Chronic dust**	***C* decreased** ***V* decreased**	***C_inf_* decreased** ***K* decreased**	***C_def_* decreased** **Hysteresis decreased**	**C57BL/6**	**M**	**NPV**	**20 and 40 cmH_2_O**	**5 and 20 BPM**	**This work**

† Reported as changes in exposed groups compared with control groups.

BPM, breaths per minute; NPV, negative-pressure ventilation; PPE, porcine pancreatic elastase; PPV, positive-pressure ventilation.

## Data Availability

Data will be made available upon reasonable request.

## References

[1] Lerios T, Knopp JL, Holder-Pearson L, Guy EFS, Chase JG. An identifiable model of lung mechanics to diagnose and monitor COPD. Comput Biol Med 152: 106430, 2023. doi:10.1016/j.compbiomed.2022.106430. 36543001

[2] Labaki WW, Han MK. Chronic respiratory diseases: a global view. Lancet Respir Med 8: 531–533, 2020. doi:10.1016/S2213-2600(20)30157-0. 32526184 PMC8034823

[3] Poole JA, Alexis NE, Parks C, MacInnes AK, Gentry-Nielsen MJ, Fey PD, Larsson L, Allen-Gipson D, Von Essen SG, Romberger DJ. Repetitive organic dust exposure in vitro impairs macrophage differentiation and function. J Allergy Clin Immunol 122: 375–382, 382. e1–4, 2008. doi:10.1016/j.jaci.2008.05.023. 18585769 PMC2685162

[4] Suki B, Bates JHT. Extracellular matrix mechanics in lung parenchymal diseases. Respir Physiol Neurobiol 163: 33–43, 2008. doi:10.1016/j.resp.2008.03.015. 18485836 PMC2666313

[5] Vogelmeier CF, Criner GJ, Martínez FJ, Anzueto A, Barnes PJ, Bourbeau J, Celli BR, Chen R, Decramer M, Fabbri LM, Frith P, Halpin DMG, Varela MVL, Nishimura M, Roche N, Rodríguez-Roisin R, Sin DD, Singh D, Stockley R, Vestbo J, Wedzicha JA, Agustí A. Global Strategy for the Diagnosis, Management, and Prevention of Chronic Obstructive Lung Disease 2017 Report: GOLD Executive Summary [Erratum in Arch Bronconeumol 53: 411–412, 2017]. Arch Bronconeumol 53: 128–149, 2017. doi:10.1164/rccm.201701-0218PP. 28274597

[6] Poole JA, Zamora-Sifuentes JL, De Las Vecillas L, Quirce S. Respiratory diseases associated with organic dust exposure. J Allergy Clin Immunol Pract 12: 1960–1971, 2024. doi:10.1016/j.jaip.2024.02.022. 38423290 PMC11316665

[7] Peng C, Yan Y, Li Z, Jiang Y, Cai Y. Chronic obstructive pulmonary disease caused by inhalation of dust: a meta-analysis: a meta-analysis. Medicine Baltimore 99: e21908, 2020. doi:10.1097/MD.0000000000021908. 32846856 PMC7447387

[8] Copot D, De Keyser R, Derom E, Ionescu C. Structural changes in the COPD lung and related heterogeneity. PLoS One 12: e0177969, 2017. doi:10.1371/journal.pone.0177969. 28542377 PMC5444650

[9] Nelson TM, Mariano CA, Ramirez GO, Badrou A, Quiros KAM, Shankel M, Eskandari M. Lung mechanics: material characterization of pulmonary constituents for an experimentally informed computational pipeline. Curr Protoc 4: e70001, 2024. doi:10.1002/cpz1.70001. 39240156

[10] Biselli PJC, Benini Kohler J, Righetti R, de Fátima Lopes Calvo Tibério I, de Arruda Martins M, Degobbi Tenorio Quirino dos Santos Lopes F. Analysis of respiratory mechanics in animal models: its use in understanding lung behavior in emphysema and asthma. Drug Discov Today Dis Models 29-30: 11–17, 2019. doi:10.1016/j.ddmod.2019.10.001.

[11] Ghorani V, Boskabady MH, Khazdair MR, Kianmeher M. Experimental animal models for COPD: a methodological review. Tob Induc Dis 15: 25, 2017. doi:10.1186/s12971-017-0130-2. 28469539 PMC5414171

[12] Suki B, Bartolák-Suki E, Rocco PRM. Elastase-induced lung emphysema models in mice. Methods Mol Biol 1639: 67–75, 2017. doi:10.1007/978-1-4939-7163-3_7. 28752447

[13] Beydon L, Svantesson C, Brauer K, Lemaire F, Jonson B. Respiratory mechanics in patients ventilated for critical lung disease. Eur Respir J 9: 262–273, 1996 [Erratum in Eur Respir J 10: 2692, 1997]. doi:10.1183/09031936.96.09020262. 8777962

[14] Menzies R, Gibbons W, Goldberg P. Determinants of weaning and survival among patients with COPD who require mechanical ventilation for acute respiratory failure. Chest 95: 398–405, 1989. doi:10.1378/chest.95.2.398. 2914493

[15] Johns DP, Walters JAE, Walters EH. Diagnosis and early detection of COPD using spirometry. J Thorac Dis 6: 1557–1569, 2014. doi:10.3978/j.issn.2072-1439.2014.08.18. 25478197 PMC4255165

[16] King TE Jr, Pardo A, Selman M. Idiopathic pulmonary fibrosis. Lancet 378: 1949–1961, 2011. doi:10.1016/S0140-6736(11)60052-4. 21719092

[17] Stenqvist O, Odenstedt H, Lundin S. Dynamic respiratory mechanics in acute lung injury/acute respiratory distress syndrome: research or clinical tool? Curr Opin Crit Care 14: 87–93, 2008. doi:10.1097/MCC.0b013e3282f3a166. 18195632

[18] Limjunyawong N, Fallica J, Horton MR, Mitzner W. Measurement of the pressure-volume curve in mouse lungs. J Vis Exp 95: 52376, 2015. doi:10.3791/52376. PMC4354562 25651276

[19] Robichaud A, Fereydoonzad L, Limjunyawong N, Rabold R, Allard B, Benedetti A, Martin JG, Mitzner W. Automated full-range pressure-volume curves in mice and rats. J Appl Physiol 123: 746–756, 2017. doi:10.1152/japplphysiol.00856.2016. 28751375 PMC5668446

[20] Devos FC, Maaske A, Robichaud A, Pollaris L, Seys S, Lopez CA, Verbeken E, Tenbusch M, Lories R, Nemery B, Hoet PH, Vanoirbeek JA. Forced expiration measurements in mouse models of obstructive and restrictive lung diseases. Respir Res 18: 123, 2017. doi:10.1186/s12931-017-0610-1. 28629359 PMC5477381

[21] Sattari S, Mariano CA, Kuschner WG, Taheri H, Bates JHT, Eskandari M. Positive- and negative-pressure ventilation characterized by local and global pulmonary mechanics. Am J Respir Crit Care Med 207: 577–586, 2023. doi:10.1164/rccm.202111-2480OC. 36194677 PMC10870900

[22] Engelberts D, Malhotra A, Butler JP, Topulos GP, Loring SH, Kavanagh BP. Relative effects of negative versus positive pressure ventilation depend on applied conditions. Intensive Care Med 38: 879–885, 2012. doi:10.1007/s00134-012-2512-5. 22349427 PMC3463870

[23] Dong SJ, Wang L, Chitano P, Coxson HO, Vasilescu DM, Paré PD, Seow CY. Lung resistance and elastance are different in ex vivo sheep lungs ventilated by positive and negative pressures. Am J Physiol Lung Cell Mol Physiol 322: L673–L682, 2022. doi:10.1152/ajplung.00464.2021. 35272489

[24] Quiros KAM, Nelson TM, Ulu A, Dominguez EC, Biddle TA, Lo DD, Nordgren TM, Eskandari M. A comparative study of ex-vivo murine pulmonary mechanics under positive- and negative-pressure ventilation. Ann Biomed Eng 52: 342–354, 2024. doi:10.1007/s10439-023-03380-1. 37906375 PMC10808462

[25] Sattari S, Mariano CA, Vittalbabu S, Velazquez JV, Postma J, Horst C, Teh E, Nordgren TM, Eskandari M. Introducing a custom-designed volume-pressure machine for novel measurements of whole lung organ viscoelasticity and direct comparisons between positive- and negative-pressure ventilation. Front Bioeng Biotechnol 8: 578762, 2020. doi:10.3389/fbioe.2020.578762. 33195138 PMC7643401

[26] Quiros KAM, Nelson TM, Sattari S, Mariano CA, Ulu A, Dominguez EC, Nordgren TM, Eskandari M. Mouse lung mechanical properties under varying inflation volumes and cycling frequencies. Sci Rep 12: 7094, 2022. doi:10.1038/s41598-022-10417-3. 35501363 PMC9059689

[27] Dominguez EC, Phandthong R, Nguyen M, Ulu A, Guardado S, Sveiven S, Talbot P, Nordgren TM. Aspirin-triggered resolvin D1 reduces chronic dust-induced lung pathology without altering susceptibility to dust-enhanced carcinogenesis. Cancers (Basel) 14: 1900, 2022. doi:10.3390/cancers14081900. 35454807 PMC9032113

[28] Vanoirbeek JAJ, Rinaldi M, De Vooght V, Haenen S, Bobic S, Gayan-Ramirez G, Hoet PHM, Verbeken E, Decramer M, Nemery B, Janssens W. Noninvasive and invasive pulmonary function in mouse models of obstructive and restrictive respiratory diseases. Am J Respir Cell Mol Biol 42: 96–104, 2010. doi:10.1165/rcmb.2008-0487OC. 19346316

[29] Takeuchi M, Sedeek KA, Schettino GPP, Suchodolski K, Kacmarek RM. Peak pressure during volume history and pressure–volume curve measurement affects analysis. Am J Respir Crit Care Med 164: 1225–1230, 2001. doi:10.1164/ajrccm.164.7.2101053. 11673214

[30] Hughes R, May AJ, Widdicombe JG. Stress relaxation in rabbits’ lungs. J Physiol 146: 85–97, 1959. doi:10.1113/jphysiol.1959.sp006179. 13655217 PMC1356891

[31] Hildebrandt J. Dynamic properties of air-filled excised cat lung determined by liquid plethysmograph. J Appl Physiol 27: 246–250, 1969. doi:10.1152/jappl.1969.27.2.246. 5796316

[32] Ramirez GO, Mariano CA, Carter D, Eskandari M. Visceral pleura mechanics: Characterization of human, pig, and rat lung material properties. Acta Biomater 189: 388–398, 2024. doi:10.1016/j.actbio.2024.09.003. 39251049

[33] Baggio C, Velazquez JV, Fragai M, Nordgren TM, Pellecchia M. Therapeutic targeting of MMP-12 for the treatment of chronic obstructive pulmonary disease. J Med Chem 63: 12911–12920, 2020. doi:10.1021/acs.jmedchem.0c01285. 33107733 PMC8544245

[34] Nelson TM, Quiros KAM, Dominguez EC, Ulu A, Nordgren TM, Nair MG, Eskandari M. Healthy and diseased tensile mechanics of mouse lung parenchyma. Results Eng 22: 102169, 2024. doi:10.1016/j.rineng.2024.102169.

[35] GOLD Report. Chapter 1. Definition and Overview (Online). https://goldcopd.org/wp-content/uploads/2021/05/GOLD-2021-Chapter-1.pdf [2025 Jan 15].

[36] Schenker M. Exposures and health effects from inorganic agricultural dusts. Environ Health Perspect 108, Suppl 4: 661–664, 2000. doi:10.1289/ehp.00108s4661. 10931784 PMC1637665

[37] Nelson TM, Quiros KAM, Dominguez EC, Ulu A, Nordgren TM, Eskandari M. Diseased and healthy murine local lung strains evaluated using digital image correlation. Sci Rep 13: 4564, 2023. doi:10.1038/s41598-023-31345-w. 36941463 PMC10026788

[38] Mandal S, Balas VE, Shaw RN, Ghosh A. Prediction analysis of idiopathic pulmonary fibrosis progression from OSIC dataset. 2020 IEEE International Conference on Computing, Power and Communication Technologies (GUCON). Greater Noida, India, 2020, p. 861–865. doi:10.1109/GUCON48875.2020.9231239.

[39] Badrou A, Mariano CA, Ramirez GO, Shankel M, Rebelo N, Eskandari M. Towards constructing a generalized structural 3D breathing human lung model based on experimental volumes, pressures, and strains. PLoS Comput Biol 21: e1012680, 2025. doi:10.1371/journal.pcbi.1012680. 39804822 PMC11729960

[40] Zosky GR, Janosi TZ, Adamicza A, Bozanich EM, Cannizzaro V, Larcombe AN, Turner DJ, Sly PD, Hantos Z. The bimodal quasistatic and dynamic elastance of the murine lung. J Appl Physiol 105: 685–692, 2008. doi:10.1152/japplphysiol.90328.2008. 18556435

[41] Nelson TM, Quiros KAM, Mariano CA, Sattari S, Ulu A, Dominguez EC, Nordgren TM, Eskandari M. Associating local strains to global pressure-volume mouse lung mechanics using digital image correlation. Physiol Rep 10: e15466, 2022. doi:10.14814/phy2.15466. 36207795 PMC9547081

[42] Sattari S, Mariano CA, Eskandari M. Biaxial mechanical properties of the bronchial tree: Characterization of elasticity, extensibility, and energetics, including the effect of strain rate and preconditioning. Acta Biomater 155: 410–422, 2023. doi:10.1016/j.actbio.2022.10.047. 36328122

[43] Ask K, Labiris R, Farkas L, Moeller A, Froese A, Farncombe T, McClelland GB, Inman M, Gauldie J, Kolb MRJ. Comparison between conventional and “clinical” assessment of experimental lung fibrosis. J Transl Med 6: 16, 2008. doi:10.1186/1479-5876-6-16. 18402687 PMC2365932

[44] Bayliss LE, Robertson GW. The visco-elastic properties of the lungs. Exp Physiol 29: 27–47, 1939. doi:10.1113/expphysiol.1939.sp000792.

[45] Boucher M, Henry C, Khadangi F, Dufour-Mailhot A, Tremblay-Pitre S, Fereydoonzad L, Brunet D, Robichaud A, Bossé Y. Effects of airway smooth muscle contraction and inflammation on lung tissue compliance. Am J Physiol Lung Cell Mol Physiol 322: L294–L304, 2022. doi:10.1152/ajplung.00384.2021. 34936511

[46] Chung J, Lachapelle K, Wener E, Cartier R, De Varennes B, Fraser R, Leask RL. Energy loss, a novel biomechanical parameter, correlates with aortic aneurysm size and histopathologic findings. J Thorac Cardiovasc Surg 148: 1082–1088, 2014. doi:10.1016/j.jtcvs.2014.06.021. 25129601

[47] Mead J, Whittenberger JL, Radford EP Jr. Surface tension as a factor in pulmonary volume-pressure hysteresis. J Appl Physiol 10: 191–196, 1957. doi:10.1152/jappl.1957.10.2.191. 13428643

[48] de Winter JCF. Using the Student’s t-test with extremely small sample sizes. PARE 18: 10, 2013. doi:10.7275/e4r6-dj05.

[49] Suki B, Sato S, Parameswaran H, Szabari MV, Takahashi A, Bartolák-Suki E. Emphysema and mechanical stress-induced lung remodeling. Physiology (Bethesda) 28: 404–413, 2013. doi:10.1152/physiol.00041.2013. 24186935 PMC3858211

[50] Abughanam N, Gaben SSM, Chowdhury MEH, Khandakar A. Investigating the effect of materials and structures for negative pressure ventilators suitable for pandemic situation. Emergent Mater 4: 313–327, 2021. doi:10.1007/s42247-021-00181-x. 33821231 PMC8012748

[51] Raymondos K, Molitoris U, Capewell M, Sander B, Dieck T, Ahrens J, Weilbach C, Knitsch W, Corrado A. Negative- versus positivepressure ventilation in intubated patients with acute respiratory distress syndrome. Crit Care 16: R37, 2012. doi:10.1186/cc11216. 22386062 PMC3681349

[52] Eskandari M, Sattari S, Mariano CA. Investigating the mechanics of positive- versus negative-pressure ventilation. Am J Respir Crit Care Med 203: A4671, 2021. doi:10.1164/ajrccm-conference.2021.203.1_MeetingAbstracts.A4671. PMC10870900 36194677

[53] Eskandari M, Sattari S, Quiros K. The role of interspecies variability on positive- versus negative-pressure ventilation mechanics. Am J Respir Crit Care Med 207: A5776, 2023. doi:10.1164/ajrccm-conference.2023.207.1_MeetingAbstracts.A5776. PMC10870900 36194677

[54] Hantos Z, Adamicza A, Jánosi TZ, Szabari MV, Tolnai J, Suki B. Lung volumes and respiratory mechanics in elastase-induced emphysema in mice. J Appl Physiol 105: 1864–1872, 2008. doi:10.1152/japplphysiol.90924.2008. 18845778 PMC2612464

[55] Helm E, Talakoub O, Grasso F, Engelberts D, Alirezaie J, Kavanagh BP, Babyn P. Use of dynamic CT in acute respiratory distress syndrome (ARDS) with comparison of positive and negative pressure ventilation. Eur Radiol, 19: 50–57, 2009. doi:10.1007/s00330-008-1105-8. 18651149

[56] Dong S-J, Wang L, Chitano P, Vasilescu DM, Paré PD, Seow CY. Airway and parenchymal tissue resistance and elastance in ex vivo sheep lungs: effects of bronchochallenge and deep inspiration. Am J Physiol Lung Cell Mol Physiol 322: L882–L889, 2022. doi:10.1152/ajplung.00033.2022. 35537098

[57] Robichaud A, Fereydoonzad L, Collins SL, Loube JM, Ishii Y, Horton MR, Martin JG, Mitzner W. Airway compliance measurements in mouse models of respiratory diseases. Am J Physiol Lung Cell Mol Physiol 321: L204–L212, 2021. doi:10.1152/ajplung.00470.2020. 34009049 PMC8321862

[58] Pelosi P, Gattinoni L. Respiratory mechanics in ARDS: a siren for physicians? Intensive Care Med 26: 653–656, 2000. doi:10.1007/s001340051227. 10945378

[59] De Langhe E, Vande Velde G, Hostens J, Himmelreich U, Nemery B, Luyten FP, Vanoirbeek J, Lories RJ. Quantification of lung fibrosis and emphysema in mice using automated micro-computed tomography. PLoS One 7: e43123, 2012. doi:10.1371/journal.pone.0043123. 22912805 PMC3418271

[60] Nieszkowska A, Lu Q, Vieira S, Elman M, Fetita C, Rouby J-J. Incidence and regional distribution of lung overinflation during mechanical ventilation with positive end-expiratory pressure. Crit Care Med 32: 1496–1503, 2004. doi:10.1097/01.ccm.0000130170.88512.07. 15241094

[61] Manali ED, Moschos C, Triantafillidou C, Kotanidou A, Psallidas I, Karabela SP, Roussos C, Papiris S, Armaganidis A, Stathopoulos GT, Maniatis NA. Static and dynamic mechanics of the murine lung after intratracheal bleomycin. BMC Pulm Med 11: 33, 2011. doi:10.1186/1471-2466-11-33. 21627835 PMC3128859

[62] Nordgren TM, Bauer CD, Heires AJ, Poole JA, Wyatt TA, West WW, Romberger DJ. Maresin-1 reduces airway inflammation associated with acute and repetitive exposures to organic dust. Transl Res 166: 57–69, 2015. doi:10.1016/j.trsl.2015.01.001. 25655838 PMC4458456

[63] Suki B, Bates JHT. Lung tissue mechanics as an emergent phenomenon. J Appl Physiol (1985) 110: 1111–1118, 2011. doi:10.1152/japplphysiol.01244.2010. 21212247 PMC3075131

[64] Eskandari M, Arvayo AL, Levenston ME. Mechanical properties of the airway tree: heterogeneous and anisotropic pseudoelastic and viscoelastic tissue responses. J Appl Physiol 125: 878–888, 2018. doi:10.1152/japplphysiol.00090.2018. 29745796

[65] Oliveira CLN, Araújo AD, Bates JHT, Andrade JS, Suki B. entropy production and the pressure–volume curve of the lung. Front Physiol 7: 73, 2016. doi:10.3389/fphys.2016.00073. 26973540 PMC4771753

[66] Sarkar M, Notbohm J. Evolution of force chains explains the onset of strain stiffening in fiber networks. J Appl Mech 89: 111008, 2022. doi:10.1115/1.4055586.

[67] Cereda M, Xin Y, Emami K, Huang J, Rajaei J, Profka H, Han B, Mongkolwisetwara P, Kadlecek S, Kuzma NN, Pickup S, Kavanagh BP, Deutschman CS, Rizi RR. Positive end-expiratory pressure increments during anesthesia in normal lung result in hysteresis and greater numbers of smaller aerated airspaces. Anesthesiology 119: 1402–1409, 2013. doi:10.1097/ALN.0b013e3182a9b0c1. 24025616 PMC3987989

[68] Mariano CA, Sattari S, Ramirez GO, Eskandari M. Effects of tissue degradation by collagenase and elastase on the biaxial mechanics of porcine airways. Respir Res 24: 105, 2023. doi:10.1186/s12931-023-02376-8. 37031200 PMC10082978

[69] Sattari S, Eskandari M. Characterizing the viscoelasticity of extra- and intra-parenchymal lung bronchi. J Mech Behav Biomed Mater 110: 103824, 2020. doi:10.1016/j.jmbbm.2020.103824. 32957174

[70] Yuan H, Kononov S, Cavalcante FS, Lutchen KR, Ingenito EP, Suki B. Effects of collagenase and elastase on the mechanical properties of lung tissue strips. J Appl Physiol 89: 3–14, 2000. doi:10.1152/jappl.2000.89.1.3. 10904029

[71] Brewer KK, Sakai H, Alencar AM, Majumdar A, Arold SP, Lutchen KR, Ingenito EP, Suki B. Lung and alveolar wall elastic and hysteretic behavior in rats: effects of in vivo elastase treatment. J Appl Physiol 95: 1926–1936, 2003. doi:10.1152/japplphysiol.00102.2003. 12871961

[72] Scaramuzzo G, Spinelli E, Spadaro S, Santini A, Tortolani D, Dalla Corte F, Pesenti A, Volta CA, Grasselli G, Mauri T. Gravitational distribution of regional opening and closing pressures, hysteresis and atelectrauma in ARDS evaluated by electrical impedance tomography. Crit Care 24: 622, 2020. doi:10.1186/s13054-020-03335-1. 33092607 PMC7579854

[73] Ito JT, Lourenço JD, Righetti RF, Tibério IFLC, Prado CM, Lopes F. Extracellular matrix component remodeling in respiratory diseases: what has been found in clinical and experimental studies? Cells 8: 342, 2019. doi:10.3390/cells8040342. 30979017 PMC6523091

[74] Harris RS. Pressure-volume curves of the respiratory system. Respir Care 50: 78–98, 2005. 15636647

[75] Permutt S, Martin HB. Static pressure-volume characteristics of lungs in normal males. J Appl Physiol 15: 819–825, 1960. doi:10.1152/jappl.1960.15.5.819. 13734448

[76] Sansores RH, Ramirez-Venegas A, Pérez-Padilla R, Montaño M, Ramos C, Becerril C, Gaxiola M, Paré P, Selman M. Correlation between pulmonary fibrosis and the lung pressure-volume curve. Lung 174: 315–323, 1996. doi:10.1007/BF00176190. 8843057

[77] Andreeva E, Pokhaznikova M, Lebedev A, Moiseeva I, Kuznetsova O, Degryse J-M. Spirometry is not enough to diagnose COPD in epidemiological studies: a follow-up study. NPJ Prim Care Respir Med 27: 62, 2017. doi:10.1038/s41533-017-0062-6. 29138407 PMC5686137

[78] Hirai T, McKeown KA, Gomes RF, Bates JH. Effects of lung volume on lung and chest wall mechanics in rats. J Appl Physiol 86: 16–21, 1999. doi:10.1152/jappl.1999.86.1.16. 9887108

[79] Huang K, Rabold R, Schofield B, Mitzner W, Tankersley CG. Age-dependent changes of airway and lung parenchyma in C57BL/6J mice. J Appl Physiol 102: 200–206, 2007. doi:10.1152/japplphysiol.00400.2006. 16946023

[80] Tam A, Bates JHT, Churg A, Wright JL, Man SFP, Sin DD. Sex-related differences in pulmonary function following 6 months of cigarette exposure: implications for sexual dimorphism in mild COPD. PLoS One 11: e0164835, 2016. doi:10.1371/journal.pone.0164835. 27788167 PMC5082824

